# Children’s affiliation toward peers reflected in their picture drawings

**DOI:** 10.3758/s13428-022-01924-2

**Published:** 2022-07-26

**Authors:** Asami Shinohara, Miyabi Narazaki, Tessei Kobayashi

**Affiliations:** 1 NTT Communication Science Laboratories, 2–4, Hikaridai, Seika-cho, Soraku-gun, Kyoto, 619-0237 Japan; 2 Runbini Early Childhood Education and Care Center, Fukuoka, Japan

**Keywords:** Children, Picture-drawing, Affiliation, Friendship, Social relationship

## Abstract

Previous studies have demonstrated that a picture-drawing task can be an indicator of the affiliation children have with their peers. When a child draws himself/herself along with a peer, the distance between them is assumed to represent the extent of the affiliation held by the child toward the peer: the shorter the distance is, the more affiliation the child has. However, some issues remain before the picture-drawing task is established as a way to measure children’s affiliation, including the possibility that the instructions might bias the children's responses (Thomas & Gray, 1992), and inconsistency over where to measure in the children’s drawings (e.g., Song et al., 2015). In this study, we focused on the above two issues and addressed whether the picture-drawing task can be used for measuring children’s affiliation toward peers. We conducted our study in Japanese nursery schools with 3- to 6-year-old children (*N* = 676), who drew pictures of themselves and a classmate. Teachers rated how much the children had played with the drawn peer. We found that the more a child had an affiliative relationship with a peer, the shorter the distance between the drawn child and peer was when measuring the closest points and the center between the two drawn figures. Our research sheds light on the validity of the picture-drawing task for measuring children’s affiliation.

It is important for children to build and maintain friendships with their peers. Having friendships greatly contributes to a child’s well-being (Berndt, 2004; Rubin et al., 2006); those who are well accepted by peers show less problematic behavior (Van Lier & Koot, 2010), and being frequently socially involved with peers can increase one’s social competence in group life (Santos et al., 2020; Vaughn et al., 2016). One of the underlying processes to establish a friendship with peers is holding affiliative feelings toward the peers. Children tend to display such prosocial behavior as generosity (measured by resource allocation) toward peers with whom they feel an affiliation (Sparks et al., 2017). Such prosocial behavior can be a first step to building a long-term good relationship that involves the mutual provision of material and emotional support, which is called friendship (Engelmann et al., 2019). Considering these facts, the extent to which children hold affiliation toward peers is worth investigating to understand their friendships and social status in their actual social lives. Then, how can researchers measure children’s affiliation?

Many empirical studies have been successful in measuring affiliation in children by observing children’s proximity behaviors, such as hugging or latency in approaching others (Marinović et al., 2017; Plötner et al., 2015; Wolf & Tomasello, 2020a, 2020b). Other studies have used an over-imitation task (Over & Carpenter, 2009; Watson-Jones et al., 2016) or verbal questionnaires asking about children’s feelings for others (Heiphetz et al., 2014; Sparks et al., 2017) to measure children’s affiliation. In these previous studies, the researchers examined whether experimental manipulations, such as priming by an ostracism scenario (Over & Carpenter, 2009), shared membership (Plötner et al., 2015; Sparks et al., 2017), or children’s experience of joint attention with an experimenter (Wolf & Tomasello, 2020a), would increase children’s affiliation toward a fictitious character or the experimenter. However, these methodologies seem unsuitable for measuring children’s affiliation toward their actual peers in their social lives. For example, the over-imitation task requires tools and a controlled situation, such as an experimental room, which may also be necessary when using children’s proximity behaviors as an index of their affiliation. Furthermore, the over-imitation task is designed to measure how much children want to affiliate with a model who shows them irrelevant actions (Over, 2020), so it would be necessary to train a peer as the model for the over-imitation task if we measure children’s affiliation toward peers in school. This is likely to be quite difficult and costly. Regarding verbal questions, previous studies used questions such as whether a child wanted to be friends with a peer (Heiphetz et al., 2014). Such a question cannot be used to measure affiliation toward already-known peers because they might already be friends. In addition, children could answer the question to make themselves look like good kids (i.e., a child who can be friends with anyone), since the intention of the question is obvious in this case.

Although the above methodologies could be difficult to use in measuring the affiliation that children hold toward a real peer in their social lives, a picture-drawing task seems suitable for measuring such affiliation. Previous studies suggest that children’s affiliation is reflected in their drawings (Pinto & Bombi, 2008). When a child draws himself/herself along with a peer, the distance between them is assumed to represent the extent of affiliation held by the child toward the peer: the shorter the distance, the more affiliation the child has. A picture-drawing task is “user friendly” because drawing is one of the normal and popular activities of children, making it highly familiar among children (Pinto & Bombi, 2008). Indeed, some previous studies used the picture-drawing task. Researchers who examined the impact of ostracism priming on children's motivation to affiliate with others asked children to draw themselves and their best friend on a sheet of paper soon after watching either a video containing ostracism scenes or a control video (Over, 2016; Song et al., 2015; Stengelin et al., 2021). These studies measured the distance between the two closest points of each figure along the horizontal axis (Song et al., 2015, p. 834) and found that children who watched the ostracism video drew themselves and their best friend closer together. Based on this result, they concluded that priming by ostracism scenes increased children’s motivation to affiliate with others (but see Stengelin et al., 2021 for a null result with Serbian children). Note that the picture-drawing task in these previous studies was used to measure the children’s desire to belong (Over, 2016) rather than their sense of affiliation for particular others.

The picture-drawing task has also been used to investigate how experimental manipulations affect children’s intergroup attitudes. Diesendruck and Menahem (2015) examined whether essentialism priming of Jewish Israeli children promoted an ethnic bias toward Arabs. They first presented a story that emphasized various aspects of essentialism to the children and asked them to draw pictures of Jews and Arabs. The distance between the two characters, measured with a ruler from the closest points of each character (Diesendruck & Menahem, 2015, p. 3), was larger when the children heard the essentialism story compared to a control story. In other studies, the children drew themselves and a novel outgroup member after hearing a negative message about the outgroup (Conder & Lane, 2021; Lane et al., 2020). The children first drew themselves in a small rectangle at the bottom center of the paper and then drew an outgroup member in an empty space on the same piece of paper. As the children’s implicit attitude toward the novel outgroup, the horizontal distance was measured between the children’s drawings of themselves and a novel group member (i.e., the distance between the closest points on the two drawings) (Conder & Lane, 2021, p. e680). They revealed that the negative message created children’s negative intergroup attitudes, which was inferred from the long distance between the drawings of the child and the outgroup member.

Thomas and Gray (1992) more directly found evidence that children’s affiliation toward peers was reflected in the distance between a drawn child and a drawn peer. They passed children a piece of paper on which one figure was already drawn and asked them to draw their “best friend” or “a child you do not like very much” on the paper, taking the drawn figure as themselves. The distance between the pre-drawn self-figure and each of the added figures was measured from the center of the head of the former to the tip of the nose of the latter. If a child failed to include a nose or drew a figure in profile, the center of the head was taken as the reference point for measurement (Thomas & Gray, 1992, p.1100). Children drew their best friend closer to the pre-drawn figure that represented themselves than the disliked peer, which indicates that the picture-drawing task can measure children’s affiliation toward a peer.

Although the potential of the picture-drawing task for measuring children’s affiliation toward peers has been shown by some previous studies, issues remain before the picture-drawing task can be conclusively established as such a measurement. Indeed, only Thomas and Gray (1992) have provided evidence that the distance between two drawn figures reflects the affiliation children are holding toward their peer. The first issue is that the drawing instructions given to children might bias their responses (i.e., drawing a picture). Thomas and Gray (1992) instructed the children to draw their “best friend” or “a child you do not like very much.” This instruction might have implicitly influenced their drawings, such as “best friend” reminding them of a situation where the two children played closely together. Thus, a more precisely controlled instruction is required when letting children draw pictures. Second, an inconsistency surfaces concerning where to measure in the children’s drawings; some measured the two closest points of each figure along the horizontal axis (e.g., Song et al., 2015), and others measured from the center of the head of one figure to the center of the nose/head of the other figure (Thomas & Gray, 1992). This inconsistency reflects that no study, which validates a measurement point of children’s drawings, captures the affiliation held by children toward others. Most previous studies using the picture-drawing task mentioned that the distance between a drawn child and a drawn other is relevant to their social relationship and proximity is one way to measure children’s affiliation; unfortunately, they measured the distance between drawn figures without a rational explanation that justified why their measurement was plausible to reveal children’s affiliation. Exactly where to measure in children’s drawings must be clarified.

In the current study, we addressed whether the picture-drawing task can be used to measure children’s affiliation toward their peers. We investigated whether the distance between two drawn figures would be related to children’s affiliation toward a real peer when they draw themselves and the peer, incorporating the above methodological issues. We asked 3- to 6-year-old children to draw themselves and a classmate under precisely controlled instruction to measure their affiliation. Specifically, we simply asked the children to draw themselves and one of their classmates on drawing paper and measured the distance between the two drawn children. As the affiliative feeling that the children hold toward drawn classmates in their daily lives, a teacher in each class answered how often the children had played together with the drawn peer in everyday life, since children are more likely to spend more time with peers with whom they affiliate (Estell, 2007; Chen et al., 2019). Then we analyzed whether the evaluated affiliation that children hold toward one peer in their social lives would have a relationship with the distance between the two drawn figures. With the aim of identifying the optimal measurement points for capturing children’s affiliation through the picture-drawing task, we measured the distance between two drawn figures by various methods, including the approaches from previous studies (Song et al., 2015; Thomas & Gray, 1992), and examined which measurement methods would best reflect the children’s affiliation.

## Method

### Participants

Eight hundred and thirty-two 3- to 6-year-old children from 21 nursery schools in Japan participated in this study.^1^ All participants were asked to draw pictures of themselves and one of their classmates on drawing paper. We excluded the data of 156 participants from the final sample for the following reasons: no picture was drawn (*n* = 12), only one person was drawn (*n* = 5), more than two persons were drawn (*n* = 50)^2^, the drawing paper was used in a different orientation (*n* = 7), two drawn figures were positioned up and down (not left and right; *n* = 7)^3^, the pictures were impossible to analyze (e.g., figures were not identifiable; *n* = 43), the data were missing that identified which figure in the picture represented its drawer (*n* = 4), the participant’s gender was unknown (*n* = 1), and the affiliative scores were missing (*n* = 27). Therefore, the final sample included 22 3-year-olds (eight boys, M = 3.86 years, SD = 0.15 years), 100 4-year-olds (43 boys, M = 4.56 years, SD = 0.31 years), 265 5-year-olds (125 boys, M = 5.51 years, SD = 0.33 years) and 289 6-year-olds (146 boys, M = 6.20 years, SD = 0.23 years). We collected the data from September 2020 to January 2021. This study was reviewed and approved by the NTT Communication Science Laboratories ethics committee (number: R02-011).

### Measurements

Children’s drawing task: A drawing task was conducted in each classroom. The children were asked to draw themselves and one of their classmates (irrespective of gender) on a 270-mm (vertical) ×380-mm (horizontal) piece of drawing paper. To decide who would draw whom, a teacher in each class first made a roster that listed the children’s names in a specific order (e.g., alphabetical, date of birth). The child listed first in the roster drew the child listed second, the one listed second in the roster drew the one listed third, etc. The child listed last in the roster drew the one listed first. Before the children engaged in the drawing task, the teacher told them, “Please draw yourself and XXX (the name of a classmate) on a drawing paper.” The children were also told not to interact with their peers while drawing their pictures. We also instructed the teacher to avoid commenting on the children’s drawings or interrupting them during the drawing task. We wanted the children to draw pictures freely on a blank piece of paper like Song et al. (2015). Since such a drawing method closely resembles their own daily drawing activity, this drawing task can be easily adapted not only to experimental settings but also in childcare fields if researchers or nursery schoolteachers want to measure children’s affiliation toward their peers. After a child finished the drawing, the teacher asked the child which figure in the drawing paper represented himself/herself (left or right). They wrote the child’s answers about their position on the paper (circled one of the options; left or right), as well as the child’s age (year and month) and gender on an information sheet that was on the back of the piece of drawing paper.

Teacher’s rating of affiliative relationships: We asked the teacher in each class to rate how much the drawn child in a picture had been playing with the drawn classmate (affiliative relationship: has never played, has occasionally played, has sometimes played, has often played, and has always played) based on their observations over the last 3 months.

### Procedure

Teachers conducted our study at each nursery school. Before the investigation, we explained to them how to conduct the picture-drawing task and make the affiliative relationships. To avoid teacher biases, we did not reveal the purpose of this study and asked the teachers to rate the children’s affiliative relationships without looking at their pictures. During the investigations, the teachers could reach us by e-mail or phone. Each teacher sent the children’s drawings and the affiliative relationship questionnaires to the first author after finishing all the assignments. The average time from an investigation request to returning the data was 20 days (min 11 days and max 70 days). Unfortunately, we lack exact data concerning when they conducted the picture-drawing task and rated the children’s affiliative relationships. The time intervals between the two tasks possibly influenced how representative the affiliation relationships were to the actual drawing distances. However, since friendship ties during preschool ages tend to be stable (Daniel et al., 2016; Wang et al., 2019), and 20 days was the average maximum interval between the two tasks, we assume the influence of the time intervals is negligible. After collecting all of the data, we debriefed the teachers about the purpose and results of this research.

### Coding

**Fig. 1:**
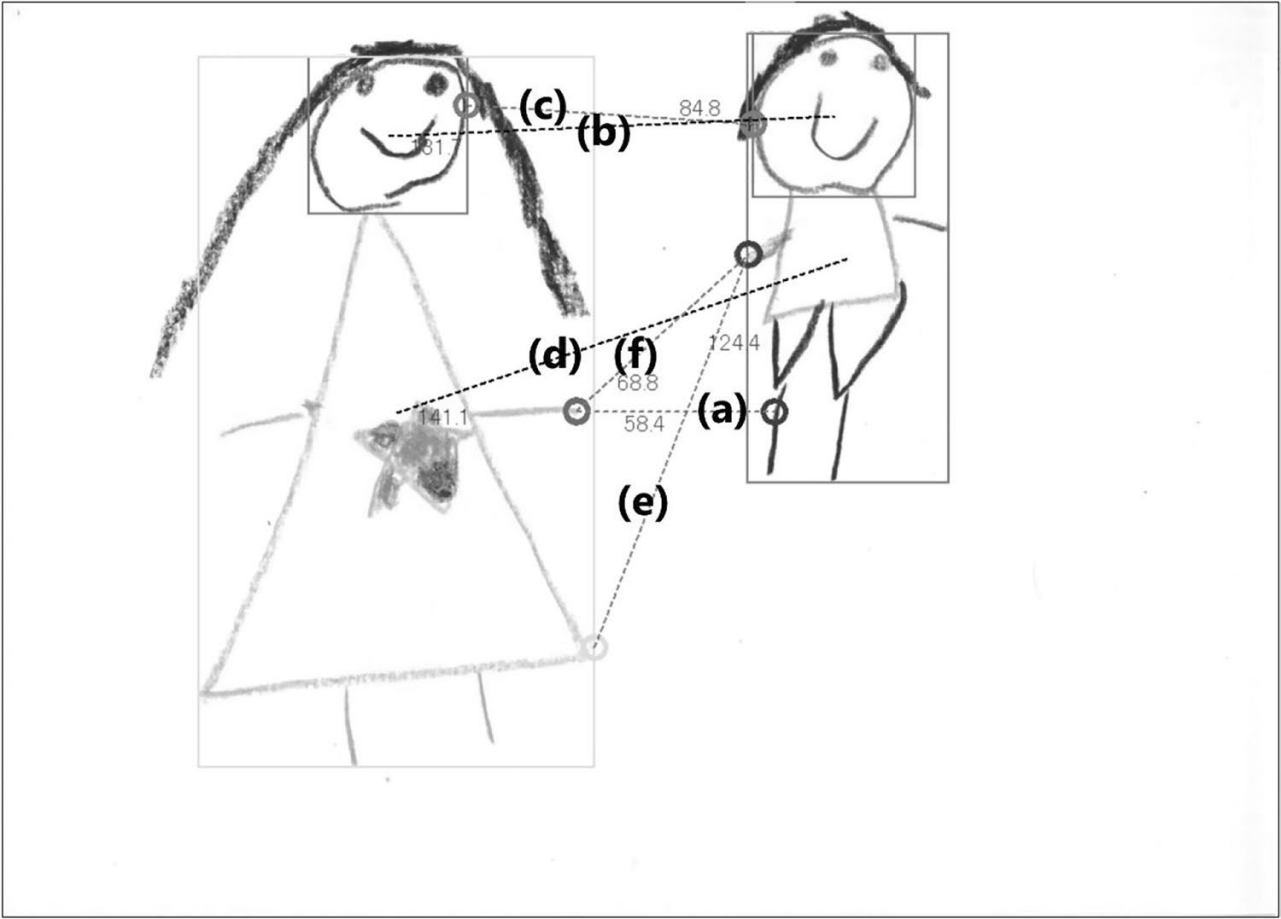
Screen of coding system. Using the CAD system, we coded the following distances: (**a**) closest point, (**b**) center of head, (**c**) edge of head, (**d**) center of figure, (**e**) edge of figure, and (**f**) hand

Children’s drawings: We measured the distance between the two drawn figures by the following six methods (all in millimeters, Fig. 1). First, we measured the closest points of each drawn figure along the horizontal axis (closest point; e.g., Song et al., 2015). If the two drawn figures overlapped or touched, the closest point was coded as 0 mm. Second, we measured the distance between the center of the head of one figure and the center of the head of another figure (center of head; Thomas & Gray, 1992). In order to determine the center of a drawn head, we drew a rectangular box around each head so that the four sides of the box touched the furthest extent of the head in each direction. Then we measured from the center of the rectangular box around one head to that of the rectangular box around the other head. Third, we measured from the edge of one head (i.e., where the side of the rectangular box and the head were in contact) to the edge of the other head (edge of head). Fourth, we measured the distance between the center of one figure and the center of another figure (center of figure). We again drew a rectangular box around each whole figure so that the four sides of the box touched the furthest extent of the whole figure in each direction to determine the center of the drawn figure including the body. After that, we measured from the center of the rectangular box around one figure to that of the rectangular box around the other figure. Fifth, we measured from the edge of one figure (i.e., where the side of the rectangular box and the figure were in contact) to the edge of the other figure (edge of figure). Finally, we measured the distance between the closest points of each drawn hand (hand). If only heads were drawn or either body had not been drawn, both the center of figure and the edge of figure were coded as “no data.” Similarly, if no hand was drawn, the hand was coded as “no data.” We measured the last four measurements for the exploratory analysis of where to measure between two drawn figures to capture the children’s affiliation since there might be a better measurement point than the closest point or the center of head, which previous studies measured (e.g., Song et al., 2015; Thomas & Gray, 1992). Furthermore, we coded the sizes of the drawn participants’ heads and their whole figures. By checking the information sheet on the backside of the drawing paper, we identified which figure was the participant himself/herself. The horizontal lengths of the rectangular boxes around a participant’s head and the whole figure of the participants were measured in millimeters. We ignored hair when coding. All the coding was done using the AR_CAD system (SHF Co., Ltd.).

Teacher’s rating of affiliative relationships: As a result of the teachers’ ratings, 172 children were coded as “has never played” with the drawn peer, 208 children were “has occasionally played,” 195 children were “has sometimes played,” 62 children were “has often played,” and 39 children were “has always played.” We coded the affiliative relationships as an affiliative score ranging from 0 (has never played) to 4 (has always played) and treated this score as an ordinal variable.

## Results

**Fig. 2:**
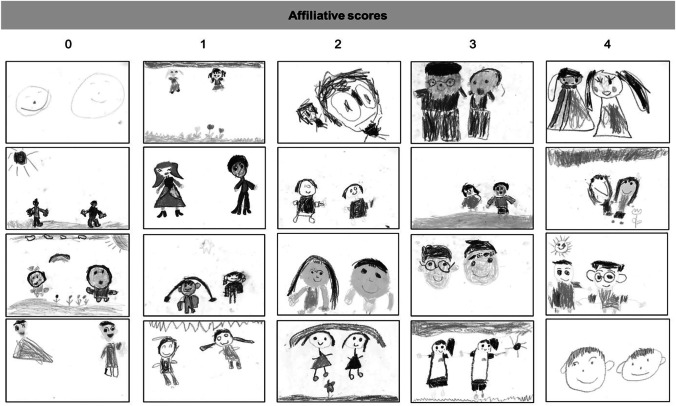
Examples of children’s drawings of themselves and a peer classified by affiliative scores (0 to 4)

Figure 2 shows examples of children’s drawings. Here, 460 children drew heads and bodies with hands of both themselves and peers, 77 children drew heads and bodies without hands, and 139 children drew only heads. The number of children who drew only heads was not significantly different depending on the affiliative scores (*χ*^*2*^ (4) = 2.05, *p* = 0.73: has never played, 35 of 172; has occasionally played, 36 of 208; has sometimes played, 46 of 195; has often played, 15 of 62; has always played, 7 of 39). In addition, the number of children who drew heads and bodies without hands was not significantly different depending on the affiliative scores (*χ*^*2*^ (4) = 1.65, *p* = 0.80; has never played, 18 of 172; has occasionally played, 23 of 208; has sometimes played, 23 of 195; has often played, 10 of 62; has always played, 3 of 39). Moreover, sometimes the two figures were not drawn on a horizon, and thus six drawings were coded as missing values, making the number of available data for the analysis of the closest point 670. The number of available data for the analysis of the center of head and edge of head was 676, the number of available data for the analyses of the center of body and edge of body was 537, and the number of available data for the analysis of hand was 460.

We ran a negative binomial model using the R package *glmmTMB* (Brooks et al., 2017) for each distance between the two figures except for the analysis of the closest point. Due to zero inflation in the distance of the closest points (*n* = 86), we used a zero-inflated model with a negative binomial error function, as in previous studies (Song et al., 2015; Stengelin et al., 2021). We entered the affiliative scores as an ordinal predictor and the participant’s age in months as a continuous variable. Because children were nested within classrooms and schools, we also entered classroom and school as random intercepts in the model. For control variables, we included the participants’ gender, the drawn peer’s gender, and the size of the drawn participant in the models. As the size of the drawn participant, we entered the size of the head when analyzing the distance between the centers of head or edges of head and the size of the drawn participant’s figure when analyzing the distance between the centers of figure, edges of figure, or hands. Regarding the analysis of the closest points, we used a maximum width of a drawn participant (either the size of head or figure, whichever was larger) as a control variable. First, we tested whether the relation between the affiliative scores (0–4) and the distance between the two drawn figures varied with age. We put the following variables into the full model: the distance as a response variable, the affiliative scores, the participant’s age (in months), the interaction between affiliative scores and age as predictors, three control variables, and the random intercepts. We tested the significance of the interaction by comparing the fit of the full model with the reduced model (without the interaction) using a likelihood ratio test. If the interaction was not significant, we omitted it and ran the reduced model. The averages of each distance, a summary of the model comparisons, and the results of all the models are found in Tables 1, 2, and 3.

**Table 1:** Averages of each distance for each affiliative score

	Affiliative scores	Mean (mm)	SD
Closest point	0: never	40.06	35.62
	1: occasionally	38.25	37.04
	2: sometimes	37.22	39.25
	3: often	39.30	35.98
	4: always	29.81	25.77
Center of head	0: never	136.50	42.61
	1: occasionally	136.45	47.50
	2: sometimes	139.75	50.62
	3: often	130.01	43.63
	4: always	126.02	41.71
Edge of head	0: never	73.08	41.78
	1: occasionally	73.95	44.14
	2: sometimes	71.25	44.38
	3: often	67.15	40.24
	4: always	69.28	36.45
Center of figure	0: never	137.04	40.46
	1: occasionally	129.25	43.08
	2: sometimes	131.45	49.55
	3: often	128.11	45.98
	4: always	118.95	42.72
Edge of figure	0: never	66.03	44.06
	1: occasionally	62.23	42.91
	2: sometimes	64.71	46.35
	3: often	62.97	36.90
	4: always	54.45	39.33
Hand	0: never	53.24	36.96
	1: occasionally	54.03	38.32
	2: sometimes	55.38	43.62
	3: often	49.12	43.16
	4: always	42.65	31.24

**Table 2:** Summary of model comparisons between full and reduced models using a likelihood ratio test

	*χ*^*2*^	*df*	*p*
Closest point	3.83	5	0.57
Center of head	2.43	4	0.66
Edge of head	1.14	4	0.89
Center of figure	2.53	4	0.64
Edge of figure	2.78	4	0.60
Hand	1.34	4	0.86

**Table 3:** Summary of generalized linear mixed models for each variable

	*Estimate* (*SE*)	*z* value	*p*
Closest point			
(Intercept)	3.86 (0.36)	10.63	< 0.001
Affiliative scores	– 0.22 (0.11)	– 1.99	0.047
Participant’s age	0.004 (0.004)	0.87	0.39
Participant’s gender (boy)	– 0.31 (0.07)	– 4.33	< 0.001
Drawn peer’s gender (boy)	– 0.00 (0.07)	– 0.01	1.00
Size of drawn self	– 0.003 (0.001)	– 3.61	< 0.001
Center of head			
(Intercept)	4.67 (0.14)	32.63	< 0.001
Affiliative scores	– 0.06 (0.04)	– 1.49	.14
Participant’s age	0.000 (0.002)	0.21	0.83
Participant’s gender (boy)	– 0.09 (0.03)	– 3.43	< 0.001
Drawn peer’s gender (boy)	– 0.01 (0.03)	– 0.56	0.57
Size of drawn self	0.003 (0.00)	9.25	< 0.001
Edge of head			
(Intercept)	4.42 (0.24)	18.18	< 0.001
Affiliative scores	– 0.09 (0.07)	– 1.20	0.23
Participant’s age	0.000 (0.003)	0.12	0.90
Participant’s gender (boy)	– 0.13 (0.05)	–2.84	< 0.01
Drawn peer’s gender (boy)	– 0.04 (0.05)	– 0.95	0.34
Size of drawn self	– 0.002 (0.001)	– 2.78	< 0.01
Center of figure			
(Intercept)	4.39 (0.17)	25.42	<0.001
Affiliative scores	– 0.09 (0.04)	– 2.00	.0046
Participant’s age	0.001 (0.002)	0.52	0.60
Participant’s gender (boy)	– 0.12 (0.03)	– 4.09	< 0.001
Drawn peer’s gender (boy)	0.02 (0.03)	– 0.85	0.40
Size of drawn self	0.003 (0.00)	11.11	< 0.001
Edge of figure			
(Intercept)	4.58 (0.36)	12.79	< 0.001
Affiliative scores	-0.08 (0.10)	-0.76	0.44
Participant’s age	-0.01 (0.005)	-1.99	0.047
Participant’s gender (boy)	-0.13 (0.07)	-1.95	0.051
Drawn peer’s gender (boy)	0.04 (0.06)	0.64	0.52
Size of drawn self	0.002 (0.001)	2.89	< 0.01
Hand			
(Intercept)	4.01 (0.46)	8.66	< 0.001
Affiliative scores	– 0.17 (0.11)	– 1.49	0.14
Participant’s age	– 0.005 (0.006)	– 0.73	0.46
Participant’s gender (boy)	– 0.22 (0.08)	– 2.78	< 0.01
Drawn peer’s gender (boy)	0.07 (0.07)	0.95	0.34
Size of drawn self	0.002 (0.001)	2.81	< 0.01

The comparisons between the full and reduced models revealed that the interaction between the affiliative scores and age did not significantly predict all six distances between the two drawn figures (Table 2). Thus, we ran the reduced model for each response variable.

**Fig. 3:**
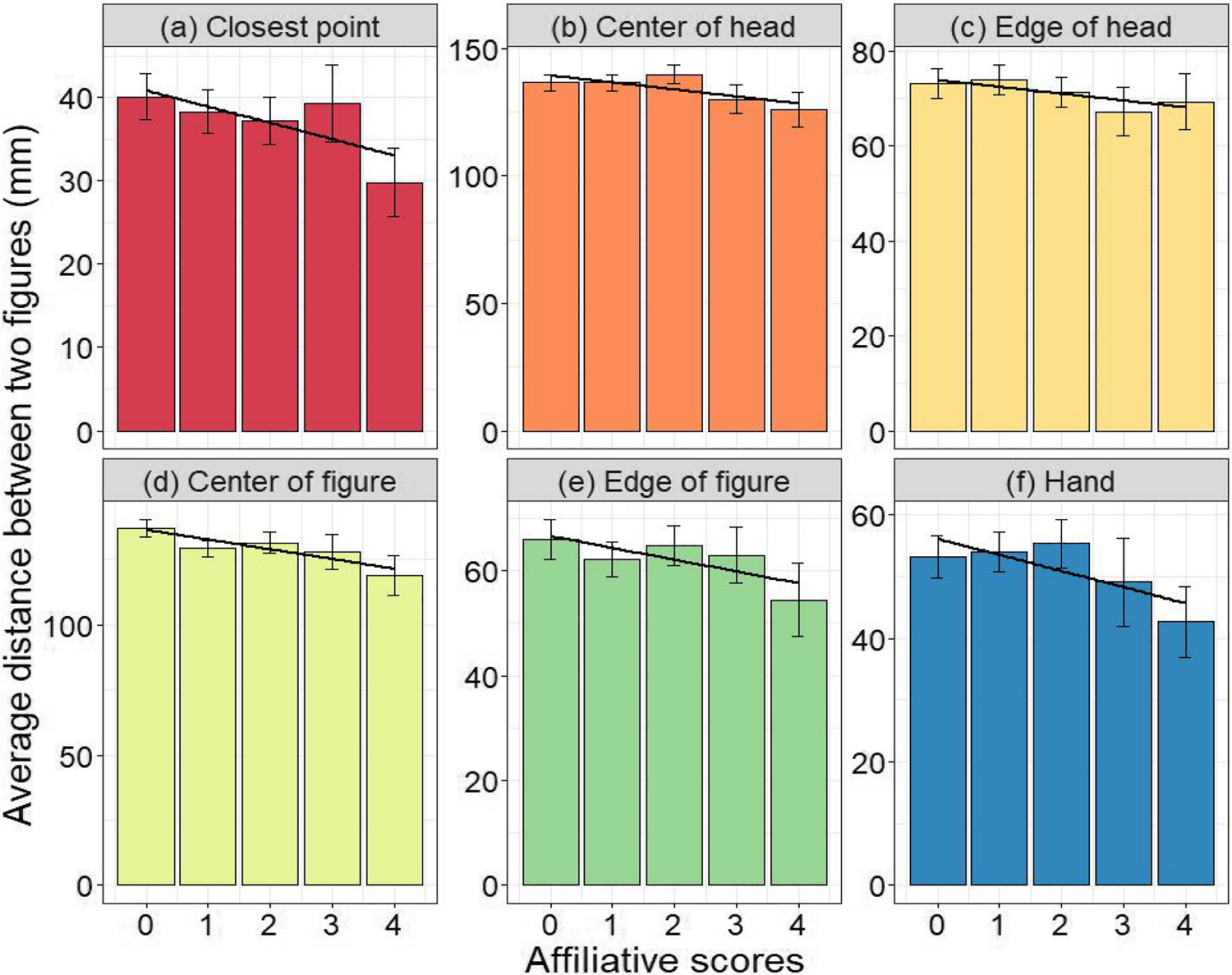
Average distances between two drawn figures (mm) for each measured point: Error bars indicate SE, and black lines indicate linear effects of affiliative scores

The model for the closest point found a significant effect of the affiliative score on the distance between the two closest points (Fig. 3a, *Estimate* = – 0.22, *p* = 0.047, *AIC* = 6029.0). As the affiliative scores increased, the distance linearly shortened between the two closest points. The model for the center of the figure also found a significant effect of the affiliative scores on the distance between the two centers of the figures (Fig. 3d, *Estimate* = – 0.086, *p* = 0.046, *AIC* = 5498.6). As the affiliative scores increased, the distance between the two centers of the figures linearly shortened. The models for the other four variables (center of head, edge of head, edge of figure, and hand) showed no significant effect of the affiliative scores on the distances between two measured points (center of head, *Estimate* = – 0.060, *p* = 0.14, Fig. 3b; edge of head, *Estimate* = – 0.086, *p* = 0.23, Fig. 3c; edge of figure, *Estimate* = – 0.076, *p* = 0.44, Fig. 3e; hand, *Estimate* = – 0.17, *p* = 0.14, Fig. 3f).

## Discussion

Our research addressed whether the picture-drawing task can capture children’s affiliation toward a real peer. Specifically, we analyzed the relationship of the distance between the drawn child himself/herself and a drawn peer and the child’s affiliation toward the peer. For children’s affiliation, we employed a rating from a relatively objective point of view: the teacher, who was familiar with the children’s daily relationships at school, rated how often the child had played with the drawn peer. Furthermore, we controlled the instructions upon which the children drew their pictures, avoiding such terms as “best friend,” “disliked person,” or “friendship” so as not to influence the drawings. Our analysis found that the greater affiliative relationship a child had with a peer, the shorter the distance between the drawn child and the child’s peer was.

Our findings provide evidence that we can measure how much affiliation a child holds toward a peer by a method of picture drawing, which is a popular and daily activity for young children. Most previous studies used the picture-drawing task to investigate how experimental manipulations (e.g., priming by ostracism or negative overheard messages) increase or decrease children’s desire to belong socially or attitudes toward fictitious outgroup members (e.g., Lane et al., 2020; Song et al., 2015). Although one study (Thomas & Gray, 1992) concluded that children’s affiliation toward peers is expressed in their drawings as the distance between two drawn figures, perhaps the instructions (i.e., draw your best friend/disliked friend) influenced their drawings. Our results, which addressed the problematic issues in previous studies, demonstrate that the picture-drawing task is valid for capturing children’s affiliation toward peers. Since our drawing task was done in a manner similar to a child's natural drawing activity (i.e., just letting children draw themselves and a peer on a blank piece of paper), researchers can use the drawing task both in experimental settings and in nursery schools; even nursery schoolteachers can exploit such task to understand children’s relationships in their classroom.

Researchers have also relied on such verbal questions as how much children want to be friends with a character (Heiphetz et al., 2014; Sparks et al., 2017) for measuring children’s affiliation toward the character. However, verbal questions might fail to capture children’s true feelings toward a peer because they are able to understand the intention of a question, which makes them behave while considering social desirability (i.e., presenting themselves as “good kids” by pretending to answer that they want to be friends with every peer). Although the picture-drawing task requires more time and material costs than just verbal questions, it can elicit children’s true affiliation with less methodological bias.

In our study, we measured the distance between two drawn figures in various ways to reveal where to measure for capturing children’s affiliation because previous studies did not use a consistent measurement method (e.g., Song et al., 2015; Thomas & Gray, 1992). As in previous studies, we found that the distance between the two closest points along the horizontal axis (e.g., Song et al., 2015) provided the best measurement for revealing children’s affiliation. Additionally, the distance between the two centers of the figures was related to their affiliation toward a drawn peer. Looking at the AIC values, the model using the center of the figure showed better fitness (*AIC* = 5498.6) than the model using the closest point (*AIC* = 6029.0). However, since approximately 20% of children did not draw complete bodies of themselves and the peer, measuring the closest points covered more drawings than the center of the figure; we can measure the children’s affiliation even when only heads are shown in the picture.

There are some limitations that must be acknowledged. First, we relied on teachers’ ratings of how much a child had played with a drawn peer (i.e., how much they spent good times with the peer) to assess affiliation toward peers. However, it might be possible that these ratings were biased by the teachers in some ways, or there might be more or less affiliative relationships between the child and the peer that the teachers were unable to observe within their classroom. Although we found that their ratings of how much the children had played with the peer were related to the distances between the two drawn figures, it may also be necessary to observe the children’s daily lives, for instance, how much they display affiliative behavior toward peers or how long they play with them, to precisely assess their affiliation. As a second limitation, we tested 3- to 6-year-old children like previous studies of children’s drawings and affiliation (e.g., Song et al., 2015; Thomas & Gray, 1992). Our study confirmed that age is not relevant to the relationship between children’s drawing and their affiliation toward peers. However, to validate the picture-drawing task for a wider age range, the data of both younger and older children are needed.

In conclusion, we provided the first evidence that suggests that the affiliation of 3- to 6-year-olds toward peers is reflected in the distance between a drawn child and a drawn peer. Our research also sheds light on the validity of the picture-drawing task for measuring the affiliation children hold toward peers in daily life.

## Data Availability

The dataset analyzed during the current study is available at OSF (https://osf.io/4n3bw/).

## Footnotes

1 We did not run a power analysis prior to the data collection. For recruiting participating nursery schools, the second author invited teachers she knew to participate in our study. Since we were not sure how many schools and children would participate in at the beginning of the study, we were unable to determine the number of participants. Instead, we conducted a post hoc power analysis and found the following powers: .90 (closest point), .91 (center of head), .92 (edge of head), .87 (center of figure), .85 (edge of figure), and .86 (hand).

2 Previous studies (Over, 2016; Song et al., 2015; Stengelin et al., 2021) measured the distance between the child himself/herself and the closest figure if children drew more than two individuals. However, we were unable to code the pictures in such a way. In our study, the teachers were simply asked to choose between the right and left options to identify (among the two figures) the child who drew himself/herself on the paper, making it impossible to completely identify the child himself/herself among more than two figures. Therefore, we excluded from the final sample participants who drew more than two figures.

3 The measurement points of the distance between two drawn figures are different in a picture in which two figures are positioned left and right or up and down. Since the latter type of picture accounted for less than 1% of the data, we excluded these data from the analysis to unify the measurement points and analysis methods.

## References

[CR1] Berndt, T. J. (2004). Children's friendships: Shifts over a half-century in perspectives on their development and their effects. *Merrill-Palmer Quarterly*, 206–223.

[CR2] Brooks ME, Kristensen K, Van Benthem KJ, Magnusson A, Berg CW, Nielsen A, Skaug H, Mächler M, Bolker BM (2017). glmmTMB balances speed and flexibility among packages for zero-inflated generalized linear mixed modeling. The R Journal.

[CR3] Chen J, Lin TJ, Justice L, Sawyer B (2019). The social networks of children with and without disabilities in early childhood special education classrooms. Journal of Autism and Developmental Disorders.

[CR4] Conder EB, Lane JD (2021). Overhearing brief negative messages has lasting effects on children’s attitudes toward novel social groups. Child Development.

[CR5] Daniel JR, Santos AJ, Antunes M, Fernandes M, Vaughn BE (2016). Co-evolution of friendships and antipathies: A longitudinal study of preschool peer groups. Frontiers in Psychology.

[CR6] Diesendruck G, Menahem R (2015). Essentialism promotes children's inter-ethnic bias. Frontiers in Psychology.

[CR7] Engelmann JM, Haux LM, Herrmann E (2019). Helping in young children and chimpanzees shows partiality towards friends. Evolution and Human Behavior.

[CR8] Estell DB (2007). Aggression, social status, and affiliation in kindergarten children: A preliminary study. Education and Treatment of Children.

[CR9] Heiphetz L, Spelke ES, Banaji MR (2014). The formation of belief-based social preferences. Social Cognition.

[CR10] Lane JD, Conder EB, Rottman J (2020). The influence of direct and overheard messages on children's attitudes toward novel social groups. Child Development.

[CR11] Marinović V, Wahl S, Träuble B (2017). “Next to you”—Young children sit closer to a person following vicarious ostracism. Journal of Experimental Child Psychology.

[CR12] Over H, Carpenter M (2009). Priming third-party ostracism increases affiliative imitation in children. Developmental Science.

[CR13] Over H (2016). The origins of belonging: Social motivation in infants and young children. Philosophical Transactions of the Royal Society B: Biological Sciences.

[CR14] Over H (2020). The social function of imitation in development. Annual Review of Developmental Psychology.

[CR15] Pinto G, Bombi AS, Milbrath C, Trautner HM (2008). Children's drawing of friendship and family relationships in different cultures. *Children's understanding and production of pictures, drawings and art*.

[CR16] Plötner M, Over H, Carpenter M, Tomasello M (2015). The effects of collaboration and minimal-group membership on children’s prosocial behavior, liking, affiliation, and trust. Journal of Experimental Child Psychology.

[CR17] Rubin, K. H., Bukowski, W., & Parker, J. (2006). Peer interactions, relationships, and groups. In W. Damon, R. Lerner, & N. Eisenberg (Eds.), handbook of child psychology: Vol. 3. *Social, emotional, and personality development* (6th ed., pp. 571–645). New York, NY: Wiley.

[CR18] Santos AJ, Daniel JR, Antunes M, Coppola G, Trudel M, Vaughn BE (2020). Changes in preschool children’s social engagement positively predict changes in social competence: A three-year longitudinal study of Portuguese children. Social Development.

[CR19] Song R, Over H, Carpenter M (2015). Children draw more affiliative pictures following priming with third-party ostracism. Developmental Psychology.

[CR20] Sparks E, Schinkel MG, Moore C (2017). Affiliation affects generosity in young children: The roles of minimal group membership and shared interests. Journal of Experimental Child Psychology.

[CR21] Stengelin R, Golubovic A, Toppe T, Over H, Haun DB (2021). Priming third-party ostracism does not lead to increased affiliation in three Serbian communities. Journal of Experimental Child Psychology.

[CR22] Thomas GV, Gray R (1992). Children's drawings of topics differing in emotional significance: Effects on placement relative to a self-drawing: A research note. Journal of Child Psychology and Psychiatry.

[CR23] van Lier PA, Koot HM (2010). Developmental cascades of peer relations and symptoms of externalizing and internalizing problems from kindergarten to fourth-grade elementary school. Development and Psychopathology.

[CR24] Vaughn BE, Santos AJ, Monteiro L, Shin N, Daniel JR, Krzysik L, Pinto A (2016). Social engagement and adaptive functioning during early childhood: Identifying and distinguishing among subgroups differing with regard to social engagement. Developmental Psychology.

[CR25] Wang Y, Palonen T, Hurme TR, Kinos J (2019). Do you want to play with me today? Friendship stability among preschool children. European Early Childhood Education Research Journal.

[CR26] Watson-Jones RE, Whitehouse H, Legare CH (2016). In-group ostracism increases high-fidelity imitation in early childhood. Psychological Science.

[CR27] Wolf W, Tomasello M (2020). Watching a video together creates social closeness between children and adults. Journal of Experimental Child Psychology.

[CR28] Wolf W, Tomasello M (2020). Human children, but not great apes, become socially closer by sharing an experience in common ground. Journal of Experimental Child Psychology.

